# Covid-19: implications for insurer risk management and the insurability of pandemic risk

**DOI:** 10.1057/s10713-020-00054-z

**Published:** 2020-09-22

**Authors:** Andreas Richter, Thomas C. Wilson

**Affiliations:** 1 Ludwig-Maximilians-Universität München (LMU Munich), Munich, Germany; 2 Present Address: Allianz Ayudhya, Bangkok, Thailand

**Keywords:** Financial crisis, Covid-19, Pandemic risk, Resiliency, Insurability, Risk management, G01, G22, G32, H12

## Abstract

This paper analyzes the insurability of pandemic risk and outlines how underwriting policies and scenario analysis are used to build resilience upfront and plan contingency actions for crisis scenarios. It then summarizes the unique “lessons learned” from the Covid-19 crisis by baselining actual developments against a reasonable, pre-Covid-19 pandemic scenario based on the 2002 SARS epidemic and 1918 Spanish influenza pandemic. Actual developments support the pre-Covid-19 hypothesis that financial market developments dominate claims losses due to the demographics of pandemics and other factors. However, Covid-19 “surprised” relative to the pre-Covid-19 scenario in terms of its impact on the real economy as well as on the property and casualty segment as business interruption property triggers and exclusions are challenged, something that may adversely impact the insurability of pandemics as well as the perception of the industry for some time to come. The unique lessons of Covid-19 reinforce the need for resilience upfront in solvency and liquidity, the need to improve business interruption wordings and re-underwrite the book, and the recognition that business interruption caused by pandemics may not be an insurable risk due to its large accumulation potential and the threat of external moral hazard. These insurability limitations lead to a discussion about the structure and financing of protection against the impact of future pandemics.

## Introduction

Dramatic events with severe consequences for the financial sector occur with a reasonably high frequency; a non-exhaustive list of examples includes the 2011 European sovereign debt crisis, 2008 financial crisis, 2002 SARS epidemic, 2001 dotcom bubble, 1997–1998 Asian-Russian crisis, and 1987 Black Monday. Notwithstanding their frequency, each event or crisis has a unique origin, a unique evolutionary path and a unique denouement in terms of its impact on the insurance industry and society more broadly. Because of their uniqueness, insurers and regulators often adapt their risk management and supervisory frameworks following a crisis to reflect the unique “lessons learned” from the latest experience.

Most recently, Covid-19 has evolved into a crisis of global proportions with a significant impact on individual health, real economic activity, financial markets, and political and social systems; we are still at the beginning of the crisis, with a very uncertain trajectory and even more uncertainty regarding the ultimate consequences for insurers, regulation, and how society views the industry.

The goal of this paper is, first, to determine how well insurer risk management practices have worked during the crisis and, second, to speculate how risk management practices, regulatory ⁄ supervisory approaches and societal views of the industry may evolve as a consequence; these questions are important both for practitioners who strive to improve internal risk management practices based on the unique “lessons learned” as well as for prudential and systematic regulators in the design of future risk-based capital and stress testing regimes, especially to prevent systemic externalities. Third, this paper addresses the question of whether pandemics are an insurable risk and how future pandemic coverage might be affected by Covid-19. Because it is the early stage of the crisis, we are forced to speculate on future developments; detailed descriptions of the aftermath will surely follow.

Insurance risk managers have some important tools within their Enterprise Risk Management (ERM) framework to manage potential crisis scenarios (see, e.g., Wilson 2015, 2013). The most important of these include underwriting and loss accumulation limitations at the policy level and scenario analysis (also see Schoemaker 1995) to assess and manage the resiliency to complex accumulations of risks. Both are focused on building resiliency by creating favorable initial conditions as well as the optionality to exercise effective contingency measures.^1^

Pre-Covid-19 scenario analysis predictions were met to a great extent by the actual crisis in the materiality of losses from financial markets, in the relative immaterial impact from life claims, and in the effectiveness of business continuity measures. The actual impact on property and casualty claims was likely a “surprise” relative to pre-Covid-19 stress scenarios, however, because of accumulations in entertainment lines and because of unanticipated challenges to business interruption policy wordings and exclusions. The unique lessons from Covid-19 both reinforce the need for financial resilience and set out a new agenda for improving contracting language and accumulation control for property and casualty insurance. Also, the Covid-19 crisis has revealed that aspects of pandemic risk, such as business interruption and event cancelation caused by pandemic suppression, in their entirety exceed the limits of insurability. The un-insurability is mainly due to the large accumulation potential and the possibility of external moral hazard resulting from businesses’ pandemic coverage being taken into account when public policy decisions are made.

In Sect. 2, we discuss the insurability of pandemic risk, before Sect. 3 summarizes critical elements of an insurer’s crisis risk management framework, focusing on policy underwriting and scenario based risk accumulation management to build resilient initial conditions and contingency plans, emphasizing the importance of building resiliency. Section 4 outlines a generic pandemic scenario analysis, describing what insurance companies were concerned about regarding pandemics prior to the Covid-19 crisis. The goal of this section is to provide a “baseline” against which to measure the unique “surprises” caused by the actual Covid-19 developments. Section 5 compares actual Covid-19 developments to date against the pre-Covid-19 scenario for financial markets, property & casualty and life & health segments as well as indications for regulatory and public policy response. In the final Sect. 6, we conclude with some lessons learned for insurers’ crisis risk management practices and future pandemic risk coverage.

## On the insurability of pandemic risk

There are two major potential impediments to the insurability of pandemic risks: the first is the large potential loss accumulation and the second is the issue of potential *external moral hazard* caused by distorted incentives for public policy decisions.^2^

Epidemic and pandemic risks are characterized by accumulations potentially large enough to raise the question of insurability.^3^ Like natural disasters (such as floods, earthquakes, hurricanes) or man-made catastrophes (e.g., caused by terrorism), an epidemic can generate large accumulations of insured losses. As natural and man-made disasters typically are regionally concentrated events, insurance markets make use of regional diversification via global underwriting, reinsurance or insurance-linked securities in order to provide cover. (Still, certain types of such catastrophes cannot be fully covered by the insurance industry; loss scenarios provide estimates that might exceed the worldwide capacity of the industry as a whole. See, e.g., Cummins et al. 2002, for considerations regarding the markets’ capacity.)

However, pandemics by definition imply that regional diversification is likely to be less helpful for managing this threat. As demonstrated by Covid-19, a contagious virus can travel around the globe quickly, impacting a large part of the world at the same time—a characteristic which is shared by, e.g., cyber risk, where, because of the connectedness of computer systems, malware can spread around the world in very short time (a prominent example being the ransomware “WannaCry”; see, e.g., Munich Re 2017; Swiss Re 2017).^4^

While high correlation in life and health insurance potentially covered losses is a defining feature of pandemic risk, the Covid-19 crisis has highlighted the accumulation potential also with respect to financial market losses and to property and casualty losses (in particular non-property damage business interruption and event cancelation). Combined with legal uncertainty and issues with imprecise wording this has caused an unpleasant “surprise” for the industry.

Unambiguous wording and the precise definition of the insured event and coverage are straightforward requirements of an insurable risk. The consequences of the lock-down in many cases have led to discussions about whether there actually was coverage under certain policies (we will revisit this issue in more detail in Sect. 5.4). This highlights how the insurability criterion of an unambiguous and precise definition of the risk often represents a challenge for real-life insurance policies, especially when new risks or unprecedented events are concerned. The realization that, e.g., business interruption insurance might (unintendedly) include protection against losses caused by a general lock-down, resembles the problem of so-called silent cyber risk (see Guy Carpenter 2018) or, similarly, of emerging threats and unexpected court rulings in the context of liability insurance (an example is the well-known Jackson Township case; see, e.g., Kunreuther 1987).

The fact that in many cases wording has turned out to be imprecise with respect to the question of whether specifically the accumulation risk of lock-down related business interruptions was covered, suggests that these scenarios were underestimated or ignored. This also demonstrates the challenges of adequate quantification and pricing of such risk. Generally, it can be noted, that there is severe uncertainty associated with modeling the frequency and severity of a pandemic.

Another characteristic of a pandemic is the fact that, unlike most natural and man-made catastrophes, losses materialize over time. During this period, governmental and personal decisions are taken to contain or suppress the pandemic. From the insurance industry’s point of view and with future product design in mind, an important question is whether insurance coverage affects these decisions, or in other words, whether moral hazard can be an issue. As such decisions are made only after the pandemic has started, a particular focus should be on ex post moral hazard—although in the future, with now increased awareness of pandemic risk, it will also be crucial to keep an eye on the effect that the availability of protection will have on businesses’ effort in preparing for a pandemic. (However, since coverage provided by the insurance industry or through other support mechanisms, will likely be limited due to the accumulation potential, it can be expected that ex ante moral hazard will also be limited).

Incentive alignment problems can occur between the insured and the insurer, where, for instance the insured has to decide about whether or not to cancel an event. As discussed, e.g., in Turner (2020) and Clarey (2020), the 2020 Wimbledon tennis championships were canceled while the French Open were merely postponed—a difference that may well have been due to the fact that only the former had cancelation coverage. While Wimbledon’s coverage received great attention and might significantly increase the demand for pandemic insurance among event organizers, it has also highlighted the difficulties in pricing such risk and the importance of policy triggers that are beyond the insured’s control.

Defining triggers that, e.g., refer to parameters of a pandemic will not be trivial, but should become easier as in the aftermath of the Covid-19 crisis it can be expected that public response mechanisms and information collection will be reviewed and improved. However, where policy triggers involve the extent of containment measures in place, attention must be paid to a severe threat of *external moral hazard* that can result from political decision-makers’ incentives to look for deep pockets. Decisions about containment measures might be influenced by the degree to which affected businesses are insured. Even worse, there is a risk that there would be pressure on the insurance industry to cover losses even if they are excluded, or to rewrite contracts to cover business interruption retroactively. This threat is of course more likely where public perception is that policy wording has been ambiguous.

The Covid-19 crisis shows how social pressure on the industry to pay claims can become a major factor in determining the potential losses from the pandemic, potentially even if such payments require a retroactive change in terms and conditions. This pressure began in the social media with tweets from US President Trump in early March suggesting that business interruption should in any case be covered by insurers, continued with explicit action by eight US states which have introduced legislation “which would require insurers to pay claims, mainly to small businesses, despite exclusions” (Reuters 2020), and is promoted by legislators saying that “retroactive BI cover (is) needed to save US businesses” (Thind 2020). According to Walker (2020), “[i]nsurers around the world are facing legal pressure to pay business interruption claims arising from Covid-19 lockdowns, and some jurisdictions have proposed legislation to enforce retroactive coverage.”

## Crisis management

Some crises manifest linearly from trigger event to trajectory to denouement; for example, the localized crisis triggered by a hurricane has a reasonably direct evolution: the wind blows and rain falls, houses are destroyed and people dislocated, followed by localized rebuilding and recovery financed by insured claims, self-insured retentions and public contributions or relief efforts.

However, the trajectories of broader financial and social crises are often characterized by a chaotic accumulation of many things going wrong in unexpected and systemically reinforcing ways; examples such as the 2008 global financial crisis, the 2011 European sovereign debt crisis and, now, Covid-19 come to mind. Crisis management is about being resilient to both severe individual risks as well as a broad spectrum of potential unexpected and self-reinforcing risk accumulations.

Insurers have a variety of tools in their Enterprise Risk Management (ERM) framework which are useful in building resiliency and contingency actions against potential crises (see Wilson 2015, 2013). The most important tools fall into two broad categories—underwriting and accumulation management at the policy level, and internal models and scenario analysis to measure and manage more complex accumulations.

### Underwriting and accumulation control

Underwriting and accumulation control at the individual policy level are at the core of managing insurance businesses and also of crisis risk management. More specifically, *explicit and legally enforceable policy contract wordings serve to limit risks to those that are insurable and desired by the insurer as well as limiting exposure to individual insured events*.

Consider, for example, cyber insurance which presents unique underwriting challenges—the client’s exposure is difficult to assess from a risk engineering perspective, it is difficult to underwrite due to a lack of historical loss experience combined with a rapidly evolving threat landscape, and there is a high potential accumulation risk from, e.g., state-sponsored, criminal or terrorist activities. Prudent insurers underwrite cyber insurance through explicit wordings and limits on affirmative cyber contracts and, increasingly, through explicit exclusions on non-affirmative contracts are used to eliminate “silent cyber” exposures. In addition to contract terms, conditions and limits, accumulated peak exposures to individual events are often ceded to the reinsurance market under a facultative or excess of loss reinsurance cover. Collectively, these actions make the potential exposure transparent and limit the book to insurable risks within the risk appetite of the organization.

In the current pandemic risk context, this same type of structuring at the policy level is evident in the separation and limitation of property damage and non-property damage business interruption contracts. Non-property damage business interruption coverage is also challenging to underwrite due to the breadth of potential triggers, dynamically changing exposures as well as the high potential accumulations; as such, the bulk of business interruption insurance sold has property damage triggers with fewer non-property damage extensions, and these only with separate and clear limitations.

### Internal models and scenario analysis

While underwriting and accumulation control focuses on individual risks, risks can accumulate across policies and assets in complex ways. Internal models and scenario analysis are used to formulate a view on complex accumulations.

Insurers use internal models to support decision-making across a broad spectrum of activities, including the assessment of risk accumulations, the adequacy of solvency capital and the adequacy of pricing from a risk-return perspective. Value at Risk (VaR) or natural catastrophe models, capital-/earnings-/liquidity-at-risk models, and risk-based technical pricing frameworks such as Return on Risk Capital (RoRC) are important components that help fulfill these needs (see, for example, Wilson 2015).

Models work best in environments characterized by stationary dynamics and sufficient prior information to characterize potential future states and consequences. For example, financial VaR models and technical underwriting tools use historical time series and/or panel data to estimate frequency and severity distributions in the hope that past experience will be representative of potential future outcomes. Where there is insufficient data or where the environment is non-stationary yet predictable, scientific analysis and expert judgment are used to augment the data. Examples include reserve risk adjustments for lines affected by predictable but uncertain social inflation, natural catastrophe models with scientifically derived hazard maps and loss distribution assumptions for new lines of business or financial variables (see, for example, the discussion of managing known, unknown and unknowable risks in Wilson 2015).

However, useful internal models may be, they are nonetheless abstractions of a much more complex and dynamic reality. As a consequence, models work reasonably well during “normal” times, but tend to fail during times of crisis simply because the trigger, evolution and denouement of each crisis is unique with limited historical precedents. As abstractions of reality, all models will be “wrong” in the next crisis.

Prudent risk managers therefore augment internal models with scenario analysis, a process of asking “what if” by defining complex scenarios or trajectories based on the current, specific state of the world; analyzing the impact on variables of interest such as solvency, liquidity and/or earnings and reputation; evaluating these against the institution’s risk appetite; and, ultimately, taking direct actions or developing contingency plans to increase resilience. (For a detailed description of scenario analysis in the context of 2011 European sovereign debt crisis, see Wilson 2013.)

Insurers use scenario analysis and stress testing in order to enhance resilience by limiting accumulations to the potential scenario and developing potential contingency and recovery plans. Most insurers run a wide range of stress tests in order to determine whether their exposures are consistent with the institution’s risk appetite, including for example

- *Parametric* scenarios, e.g., −50/−100 bps rates down, −30% equity markets, + 50/+ 100 bps credit spreads, etc.;
- *Historical scenarios used on current exposures,* e.g., 2008 global financial crisis, 2011/2012 European sovereign debt and bank crisis, 2002 SARS or 1918 Spanish Influenza epidemics, etc.;
- *Reverse stress tests*, e.g., asking what possible scenarios would bring the company to a specific solvency or loss level?
- *Life and Health (LH) risk-specific stresses*, including mortality, morbidity or health shocks such as pandemics, increased longevity scenarios and behavioral changes to lapsation/annuitization rates for retirement products;
- *Property and casualty (PC) risk-specific stresses*, e.g., 1-in-250/500/1000 year earthquake or hurricane scenarios; maximum probable loss scenarios for cyber or man-made clashes, etc.; secondary accumulation scenarios including tsunami, wild fires, etc.;
- O*perational scenarios including business continuity* from post-catastrophe work at home conditions, failures in infrastructure, suppliers, etc.; and,
- Broader, *socio-economic stresses*, e.g., an escalation from a US–China trade war to de-globalization, a cyberwar and ultimately into limited engagement in Asia Pacific; Brexit, Italexit, Frexit, Dexit; land war on the Korean peninsula; bail-inable European bank debt during a banking crisis, etc.

While stress testing is an invaluable risk management tool, it does have its limitations: for example, just like an internal model, any stress test will be “wrong” in the details for the next crisis and it may be challenging to get management to “buy in” to stress tests based on events which are perceived as being overly extreme. Of what use to management is a scenario with a USD 500 mn potential loss but a standard deviation of USD 5 bn? Or a scenario with a USD 5 bn potential loss but a management estimated probability of occurrence of less than 0.001%, especially if management’s perceptions may be influenced by behavioral biases which restrict the recognition of extreme events (discussed in Sect. 3.3)?

Risk managers mitigate these issues through several different mechanisms: first, by comparing ad hoc stress scenarios against the results of historical stress scenarios, reinforcing the recognition that large events do in fact happen; secondly, by defining a robust risk appetite “envelope” by risk category which helps to normalize the specific details of an ad hoc scenario by using a common metric to measure the results. For example, there are many potential financial market scenarios which might lead to a USD 500 mn financial market loss (e.g., 2011/2012, 2008, 2001, 1997, etc.): the exact details of how the loss emerges is not as important as whether management has defined a “risk envelope” for financial market losses and whether the 2020 Covid-19 scenario falls within this envelope after existing resiliency measures have been tested. If it falls outside of management’s risk envelope, then further analysis and discussion are warranted.

In the context of pandemics, different elements of this stress testing framework become important: clearly, LH-specific mortality, morbidity, and longevity assumptions need to be stressed; in addition, the insurer has to take a broader view on potential systemically reinforcing accumulations or contagion to other areas, especially the impact on financial markets, potential PC claims and operational resiliency.

### Managing uncertainty: the relative importance of resiliency vs contingency plans

In lieu of a precise theoretical definition, in practice business professionals commonly distinguish between actions which build resiliency and contingency planning. The practical distinction between the two is one of timing and how the uncertainty unfolds, with actions taken to build resiliency characterized as actions taken before the risk event occurs and contingent actions taking place after the fact. For example, one business website differentiates “Risk mitigation strategies are things you can do now to reduce your company's risk (whereas a) a risk contingency plan is something you draw up now but don't deploy until the trouble comes to pass” while another differentiates “risk management (as) the practice of identifying, assessing, avoiding, mitigating, transferring, sharing and accepting (residual) risk (and) contingency planning (as) the practice of identifying steps to be taken if a risk occurs.”^5^

Under this layman’s definition, resiliency is built by positioning the firm’s financial and operational profile within management’s risk appetite so that it can continue to survive under potential future adverse events. Resiliency can be accomplished, for example, by holding additional capital and liquidity resources, building redundancy in operations to meet the potential challenges of extreme events, avoiding higher risk (but also higher expected return) investments and curtailing exposure to accumulations which may be well-priced on the margin but represent a peak accumulation outside of management’s risk appetite. Resiliency comes at a cost, however, e.g., in terms of more shareholder capital^6^ or liquidity arrangements or redundant systems, the opportunity cost of a more conservative strategic asset allocation, the cost of transferring risk via reinsurance or derivatives, etc., which may seem high during “normal” times; however, like carrying an umbrella on a sunny day, they provide security under unexpected, stormy conditions.

Contingency plans are built on top of this resilient foundation to provide another layer of protection; contingency plans can range from short-term actions (e.g., the intention to sell or hedge higher risk assets, buy reinsurance, or call on committed credit lines following a crisis, adjusting the expense base or policyholder crediting rates for retirement savings products, etc.) to more dramatic *recovery* measures (e.g., considering the disposal or run-off of businesses, fundamentally changing products, raising capital, etc., (see IAIS 2019 for a discussion of recovery planning) and, finally, to *resolution* measures which limit the impact on policyholders and society should the firm no longer be able to operate.

From a theoretical point of view, both sets of actions are the solution to a (well-defined) optimization problem: let us hypothesize that the management of an idealized insurance company maximizes a multi-period objective function as a price taker within efficient markets, subject to constraints, defining not only a set of initial positions (e.g., debt/equity ratio, cash liquidity, initial asset and liability portfolio decisions, etc.) but also state-contingent future actions to be taken as uncertainty is resolved in future states. Let us also assume that there are sufficient frictions which generate a role for risk averse decision-making (as opposed to risk neutrality), for example cost convexity driven by tax distortions or frictional costs of bankruptcy, asymmetric information between managers and shareholders and debtholders leading to signaling equilibria which give preference to actions which stabilize cash flows, risk-based capital regimes, etc. (see, for example, Smith 1998 for a good overview of why risk management may improve the value of the enterprise).

Under this stylized perspective, the representative actions under “building resilience” and “contingency planning” are both an integral part of the solution to this optimization program. This is the conclusion reached by Maes and Dann (2016) in the context of preparing for critical infrastructure catastrophes, who state, “[a]s a result, [management decisions] reflect the objective of satisfactory performance under well selected extreme conditions. The extent to which the extreme boundary is “pushed” depends on … the nature and the consequences of the hazards, and risk acceptance, all of which fit neatly into the traditional framework of decision theory. This basic framework is also broad enough to include wider socio-economic and environmental objects, so that provisions with respect to robustness, resilience, sustainability, and risk mitigative measures [including contingency plans] in general, can be effectively accounted for.”

Some of the constraints to the firm’s neo-classical optimization problem may be imposed by external stakeholders, including prudential regulators and rating agencies, for example in the form of risk-based capital regimes, stress testing requirements, etc., and many of these are binding constraints. It is challenging to distinguish resiliency actions to meet these external constraints versus an unconstrained solution reflecting only the objective function of the firm. While large insurers have been running pandemic stress tests regularly, it is not clear whether small insurers have regularly done so in the past. In any case, it is reasonable to expect that pandemic stress tests will be de rigueur for all firms in the future.

Assuming rationality of the agents leading to time consistency of the contingency actions, e.g., that it is in their best interest to execute the specific contingency action if the state of the world is achieved, and the neo-classical theory of the firm where risk management adds value, *there is no *a priori* reason to believe that current actions to promote *“resilience”* are either a better or worse management tool compared to contingent actions*—the former simply reflect the best decisions of management given the initial conditions, information and opportunity set and preferences and the latter the best decisions following an event.

#### Why resiliency is relatively more important than contingent actions

In the neo-classical model of the firm where risk management has a non-trivial role, actions which promote resiliency are important and add to the value of the firm through several channels:

- First, they help to avoid the downside of default. Prudent capital and liquidity resources aligned with a prudent risk appetite help insurers avoid frictional bankruptcy costs which dissipate value away from both shareholders and debtholders. In addition, it helps to preserve the value of their unique franchise in terms of networks, resources, and intellectual and brand capital that allows them to earn more than their cost of capital;
- Second, they open up opportunities. A strong position during a downturn allows the management of the resilient firm to take advantage of mispriced assets or competitors’ weakness (Rhodes and Stelter 2009);
- Third, contingent management actions during a crisis can have a much higher cost compared to resilience. In terms of capital structure and liquidity, firm equity is sold at a steep discount and debt is raised at a high risk premium following a crisis; and, in terms of risk reduction, risky assets are sold at a discount into illiquid markets, reinsurance is more expensive reflecting supply and demand considerations, duration lengthening comes at the cost of lower returns driven by central bank quantitative easing, etc.
- Finally, contingency plans cannot make up for lost value, which must be earned through arduous work; they can only in principle reduce the amount of required capital.

#### Why resiliency is even more important

However, there are important reasons why this stylized perspective described above may not give enough priority to resiliency versus contingency planning. From an individual firm perspective, resiliency actions may be underweighted due to behavioral biases or the inability to plan for the unimaginable or unknowable risks; from a societal perspective, a higher emphasis on resiliency may lower the probability of negative externalities from a firm or systemic failure.

#### Behavioral biases

Various behavioral factors may add to the complexity of the risk management challenge compared to the neo-classical, rational expectations solution and increase the need to focus on resiliency as opposed to contingency planning.

For example, insurance decision-makers *may underweight resiliency measures* due to a tendency to ignore information about extreme risk (Golman et al. 2017); decisions may also be affected by the availability heuristic, where decisions are influenced by readily recallable events, potentially downplaying for example a 100-year-old 1918 Spanish Influenza scenario (Tversky and Kahneman 1974); a present bias where individuals value near term rewards over more distant rewards, even above what would be implied by rational financial discounting (see, e.g., Laibson 1997; Casaburi and Willis 2018); or an optimism bias, i.e., a tendency to overestimate the probability of positive events and underestimate the probability of negative events (Sharot 2011). In addition, decision-makers may put too much emphasis on contingency plans which are not likely to be actually implemented, for instance due to inertia or a status quo bias (Samuelson and Zeckhauser 1988).

If a risk manager is aware (sophisticated) of such behavioral problems, s/he might rather (seemingly over-)invest in resiliency upfront compared to the neo-classical equilibria rather than expose him- or herself to behavioral biases in contingency planning. “Sophistication mitigates procrastination, but exacerbates preproperation” (O’Donoghue and Rabin 1999, p. 103).

#### Unknowable risks

Potentially related to the behavioral biases which lead management to downplay extreme events (e.g., the availability heuristic, the present bias or the optimism bias) is the concept of unknowable risks. The stylized economists’ perspective assumes that the space of events is known. Unknowable risks are defined as situations, “where even the events cannot be identified in advance—neither events nor probabilities are known” (Diebold et al. 2010, p. 3). Colloquially, unknowable risks are risks which are beyond imagination, including that of science fiction writers.

Unknowable risks are different from ambiguous risks which are recognized to be in the event space, but for which the probabilities are uncertain; Berger (2015) concludes that ambiguity increases the investment in self-protection.^7^ It might therefore be reasonable to hypothesize that even more fundamental uncertainty regarding the event space may likewise prompt sophisticated agents to take actions upfront which increase resilience to unknown risks, a conclusion which some authors have reached. According to Scott (2010), “[b]y definition, little can be done to learn about or manage [unknowable risks]. The timing and distribution function, or even the existence of the risk in the polar case, is a mystery. How then … should a firm cope with such possible random shocks? Primarily, by limiting its leverage and having enough capital and liquidity to absorb unknowable losses if they should occur … How much of a liquid capital margin is sufficient or appropriate for this purpose? Sufficient is, again by definition, unknowable. Appropriate is a matter of your perspective …” (Scott 2010, pp. 285–286).

#### Systemic consequences/externalities

Too little resiliency from a societal perspective may also be caused by systemic externalities or, more specifically, the consequences of individuals’ cumulative actions creating markets which are “unable to appropriately allocate resources *after* the occurrence of a surprise event or ‘shock’” (Athreya 2009).

The proposition is that individual firm defaults can have systemic implications for society similar to a cascading avalanche with financial linkages between firms triggering more frictional bankruptcy costs and leading to inefficient allocations. If resilience-building risk management activity in a firm not only helps to protect this specific firm, but also contributes to prevent the failure of other actors in the market, then such risk management has positive externalities. This will lead to underinvestment in resilience building compared to the social welfare maximizing solution (see Hofmann and Rothschild 2019).

This explains to a great extent why new financial regulation following the 2008 crisis focused on containing actions which might lead to systemic risk and increasing resilience, especially for so-called systemically important financial institutions, through higher capital requirements, greater supervisory oversight and the requirement to have recovery and resolution plans (BIS 2018).

## Setting expectations—pandemic scenario analysis, pre-Covid-19

Most pandemic scenarios used in the industry focus on novel influenza viruses; historical examples of epi novel viruses include HIV, MERS in 2012, SARS in 2002 and the 1918 Spanish influenza pandemic. They are novel in the context of human infection as they generally circulate among animals and are particularly of concern when they “jump species” and mutate to infect humans through person-to-person interaction. With no preconditioned immunities in the human population, no vaccines initially available and no antibiotics to treat secondary complications associated with the infections, control efforts are limited to non-pharmaceutical interventions such as good personal hygiene, formal or informal limitations of public interaction and rigorous testing and isolation/quarantine regimes.

Historical epidemics differ in terms of infection rates and mortality rates (see Table 1 in Chen 2020). High infection rates imply challenges to suppress the spread of the disease as well as a high potential burden on health care systems. Fortunately, some of the more recent epidemics with high mortality rates (e.g., MERS, Avian H7N9, Ebola and HIV) have relatively low infection rates, especially when combined with mitigating suppression activities. With the benefit of hindsight, the estimated 3% mortality and 1.4–5.5 infection rate of Covid-19 seems to exhibit case fatality and infection rates on par with the 1918 Spanish influenza pandemic but a lower mortality rate when compared to 2002 SARS epidemic.

**Table 1:** 1918 Spanish influenza pandemic: Fall wave mortality estimates for selected places

Continent	Deaths	Deaths per 1000
North America	0.6 million	5.3
Europe	2.3 million	4.8
Asia	19–33 million	19.7–34.2
Africa	1.9–2.3 million	14.2–17.7
Latin America	0.8–1.0 million	8.4–10.6
Pacific islands	0.09 million	–
Total	24.7–39.3 million	13.6–21.7

Table 1 in Patterson and Pyle (1991)

### Scenario impact summary

Many companies annually evaluate pandemic stress scenarios based on 2002 SARS and the 1918 Spanish Influenza, selecting SARS as a “low epidemic scenario” and the 1918 Spanish influenza as an “extreme pandemic scenario” due to its global mortality impact. In addition, many companies also ran ad hoc stress scenarios in early 2020 to analyze the emerging impact of Covid-19. Most companies reject the use of the 1346–1353 Bubonic and 542 AD Justinianic plagues as being too extreme, with a death toll of an estimated 1/3 and 1/2 of the European population, respectively (Howard 2020); these extreme historical scenarios are often rejected both because of advances in health care and sanitation but also because a relevant scenario from a managerial perspective should be extreme but not so dramatic that it cannot be survived by taking appropriate actions. Similarly, global extinction events caused by asteroids hitting the earth are generally not used for assessing insurers’ risk appetite and resiliency.

The general conclusions of these (pre-Covid-19) scenario analyses typically were that, although pandemics *may have a significant impact on human life and welfare, the losses from insurance liabilities would be immaterial relative to the losses coming from financial market developments*. This was the logical outcome given the underlying scenario assumptions, more specifically:

- The economic impact on asset and guarantee values would be material as the real economy is adversely impacted by extraordinary voluntary and forced suppression measures.^8^
- Compared to the financial market losses, life mortality and health claims would be immaterial due to
  º favorable demographics of the affected population, especially lower insurance penetration rates of many affected {age, geography, wealth}-cohorts;
  º the business mix more geared to retirement policies (longevity exposed and therefore potentially offsetting protection risks) as opposed to protection (mortality and health exposed); and,
  º finally, in terms of health care claims, the role of governments in testing and isolation activities as well as an offset due to the delay of elective or non-essential procedures.
- Property & casualty claims would likewise be immaterial compared to financial market losses due to underwriting principles which limit or exclude pandemic risks. Exceptions to this principle might occur for specific lines such as non-property business interruption (with limited historical uptake by policyholders and strict limits), entertainment/event cancelation insurance and trade credit insurance (impacted by macro-economic factors).
- Operational resilience during a pandemic would be effectively ensured through normal business continuity management activities, ensuring remote working and access by customers, distributors and staff.

In addition to using the historical SARS and Spanish influenza scenarios, some firms also ran Covid-19 stress scenarios in early 2020, coming to the same conclusions.^9^ In the remainder of this section, we outline at a high level, the SARS epidemic and 1918 Spanish influenza pandemic scenarios which firms may have used for their annual scenario analysis.

### SARS historical scenario

A good description of the SARS epidemic and resulting consequences can be found in Knobler et al. (2004). Paraphrasing Monaghan (2004), SARS had a disproportionately large economic and political impact relative to its direct costs, especially from the perspective of the insurance industry:

- *Mortality and health impact*: It is estimated that SARS caused (only) 813 deaths from 2002 to 2003. By 2003, the estimates of the infected and deaths were 8.218 and 802, respectively, for the countries most affected (China, Hong Kong, Taiwan, Singapore, Thailand and Canada).
- *The incremental impact for the insurance sector* from direct costs was limited due to a variety of factors:
  º First, the low absolute number of infected and deaths relative to other, existing covered diseases (see Figs. 5–8 in Knobler et al. 2004);
  º Second, in terms of mortality and health claims, mitigating factors for a similar scenario happening today would include
    Government provision of testing, isolation and treatment activities;
    Favorable demographics of infection and mortality, impacting geographies (e.g., a disproportionate impact on developing Asia) and age segments (e.g., a steadily increasing excess mortality curve with most fatalities in the 60+ age bracket, see Table 1 in WHO 2003) which do not typically have a high penetration rate in terms of health and mortality insurance;
    A reduction in other health care claims due to reduced non-essential procedures^10^;
  º Third, in terms of retirement investment and savings products, low mortality losses as mortality claims are materially covered by retirement account balances combined with a (potential) benefit from decreased longevity for life retirement products with in-the-money guarantees.
- *The indirect financial market impact* was triggered by a substantial decline in consumer demand which disproportionately impacted services, travel and economic activity in higher density urban areas, primarily in East Asia, as well as increased uncertainty affecting future consumption and investment. Estimates in 2003 attributed a dramatic impact on jobless rates in the region. The World Bank cut GDP estimates for East Asia by 0.8 pp and the Asian Development Bank warned that East Asia could lose USD 28 bn in output if SARS was not contained (see Table 5–1 in Knobler et al. 2004).

Taking the USD 28 bn as an (admittedly inflated) estimate of lost GDP, the extraordinary financial impact of SARS would have been on the order of USD 35 mn per fatality; when contrasted to the lack of economic losses caused by suppression activities for seasonal influenza which has a “normalized” impact of 4 mn fatalities per annum, it is fair to conclude that the impact of SARS’ extraordinary suppression activities on GDP and employment was significant.

### 1918 Spanish influenza historical scenario

The scope of infected and mortalities during the 1918 influenza pandemic clearly dwarves the 2002 SARS epidemic; more importantly, it was also felt across the globe as opposed to being primarily in Asia. Nonetheless, the implications of the scenario analyzed pre-Covid-19 would have been broadly the same: insurers would be impacted materially by financial markets, with relatively limited losses from the liability side of the balance sheet.

- *Mortality and morbidity*: According to the Center for Disease Control and Prevention (CDC 2020), “[t]he 1918 influenza pandemic was the most severe pandemic in recent history…It is estimated that about 500 million people or one-third of the world’s population became infected with this virus. The number of deaths was estimated to be at least 50 million worldwide with about 675,000 occurring in the United States.”
- Similar to the SARS scenario, in spite of the higher absolute number of infections and deaths, *the impact for the insurance sector* from direct costs was likely to have been limited due to a variety of factors:
  º First, the demographics of infections and deaths were in segments which do not typically have high penetration rates in terms of health and mortality insurance.
    With regards to *age segments*, according to the CDC (2020), “[m]ortality was high in people younger than 5 years old, 20–40 years old, and 65 years and older,” exhibiting a ‘W-shaped’ excess mortality curve which differed from the ‘U-shaped’ curve caused by normal influenza strains (Taubenberger and Morens 2006). In terms of insurance penetration, the first and last legs of the ‘W-shape’ (i.e., young children and the aged) have low mortality insurance penetration rates; while the middle leg of the ‘W’ could have increased mortality claims, the *geographic demographics* likely mitigated this effect;
    With regards to the geographic distribution, the great bulk of the mortalities in terms of absolute numbers as well as per population occurred in developing continents (Table 1) which typically have very low life and health insurance penetration rates, potentially due to an income or wealth effect^11^;
    Table 1 suggests that there is a *negative* correlation between income and infection- and mortality rates, thereby further limiting the impact on insured losses; plausible explanations for this relationship range from higher income geographies benefiting from more hygienic environments, better general health status due to diet and preventive care and fewer complicating conditions.^12^
  º Second, there are a variety of potential mitigating factors not directly documented during the 1918 Spanish Influenza but likely to be associated with a new event should it occur today:
    Government provision of testing, isolation and treatment activities;
    A reduction in other health care services and claims due to reduced utilization;
    A (potential) benefit from life retirement products due to decreased longevity and mortality claims materially covered by the retirement account balances;
- *The financial market impact* was disproportionately large. According to the stress scenarios conducted by the Congressional Budget Office to estimate the impact on the US economy of a possible pandemic (CBO 2006), “[i]n the severe pandemic scenario [roughly similar to the 1918–1919 Spanish flu outbreak], roughly 90 million people become sick and 2 million people die in the United States, and in CBO’s estimation, real GDP would be about 4–1/4% lower over the subsequent year than it would have been had the pandemic not taken place. That estimate of the effect on GDP is comparable to the effect of a typical business-cycle recession in the United States during the period since World War II.” Jonung and Roeger (2006) provide estimates of the GDP impact from a variety of different studies in their Table 4, confirming the large impact on the real economy.

The relatively low impact on mortality losses was also the conclusion based on a detailed mortality model in Stracke and Heinen (2006), who concluded that a Spanish influenza scenario applied to the German insurance market would generate “[a]dditional claims costs of nearly €5 billion–around 50% of the market's total annual gross profit (before policyholder bonuses)—[which] would strain, but surely not break, the German insurance market.” These higher claims would in many instances be offset by the reduction in the insurer’s liabilities if the coverage is provided via a retirement savings or investment policy.

## Actual developments and impact on the industry

### Actual developments

Below is a high-level assessment of where actual developments are consistent with or deviated from the pre-Covid-19 scenario.

*Financial market impacts*: Actual financial market impacts have been material and remarkably in line with the pre-Covid-19 scenario analysis; however, there has been a significant surprise in terms of the dislocation to the real economy, with the financial market effects dampened by extraordinary monetary and fiscal policy combined with expectations of a quick return to “normal.”

In terms of an outlook, it is highly likely that Fall and Winter 2020 will see an increase in credit downgrades and defaults as the latent bankruptcies caused by containment activities work their way through the system. In addition, it is also likely that further interest rate cuts will materialize after containment activities are relaxed if central banks believe that further quantitative easing may spur real economic recovery via increased demand and corporate investment. The situation could become exacerbated if a second infection wave, or “W” scenario, were to emerge (Sheiner and Yilla 2020).

*Insurance claims and operations:* Actual life and health claims and operational resiliency issues have been less material and in line with the pre-Covid-19 scenario analysis. However, actual property and casualty claims have provided the biggest surprise relative to pre-Covid-19 scenario analysis as policy wordings and exclusions are being challenged, especially for business interruption coverage, and the assumption of diversification has been critically challenged.

### Impact on valuations, solvency ratios, and capital management

None of the financial market developments have been favorable, causing lower asset values and higher economic liability valuations, especially for insurers with long-term retirement businesses. In fact, the insurance industry fared much worse than the overall stock market: over a 1 year horizon ending in early May 2020, the Eurostoxx insurance index fell −23.9% while the Eurostoxx aggregate index fell only −14.9%. This negative relative performance may seem paradoxical given the relative stability of accounting earnings for the sector; however, it can be explained by looking at market-consistent valuations as opposed to accounting earnings (see Wilson 2015, for a discussion of insurer valuations during crisis).

Financial market turbulence has also adversely impacted insurers’ solvency ratios. Focusing on European insurers under Solvency II, Deutsche Bank (2020) says that this is the “[m]ost volatile quarter on record—We estimate that market moves in 1Q20 have cost the sector c. 18 pp in aggregate, driven by lower bond yields (−8 pp), wider credit spreads and a fall in equity markets (−6 pp each) as Covid-19 spread worldwide.” Deutsche Bank estimates are in the middle of the analyst range for financial market impact, with UBS (2020) coming in at −16 pp and JPM (2020) at −25 pp impact on European insurers’ solvency ratios under Solvency II (after excluding + 2 pp of net capital generation for the industry). The solvency ratio is the amount of an insurer’s available solvency capital under Solvency II, the so-called own funds, divided by its Solvency Capital Requirement (SCR).^13^

Looking at individual company results, most of the impact on solvency came via reduced own funds as opposed to an increased SCR.^14^ In general, *management actions during a crisis can only materially reduce SCR but cannot replace lost own funds which take a long time and much effort to generate and retain, reinforcing the message that initial resilience for insurers is more powerful than contingency plans during the crisis.*

In spite of this decline, the industry remains well capitalized to meet policyholder obligations according to analysts, with analyst estimates putting the industry solvency ratio between 180% (JPM) and 190% (Deutsche Bank). UBS (2020) states, that “[d]espite high volatility, … capital buffers/ratios remain at adequate levels, within insurers' target ranges and not at levels close to impairing dividends. Given the sector is the 4th largest dividend contributor to European equities and with dividend cuts in other financial sectors—this is a key conclusion.”

### An unexpected accumulation in general insurance

In contrast to the financial market impact, the actual impact on property and casualty claims did substantially surprise relative to the pre-Covid stress scenario, shifting the focus quickly from the asset to the liability side of the balance sheet (Autonomous 2020). There are two root causes of this unexpected development: first, the interpretation of pandemic exclusions and other terms and conditions limiting pandemic risk in general insurance lines is being challenged and, second, accumulation scenarios in specialty lines did not fully anticipate the broad impact of the pandemic’s suppression activities.

Initial estimates put the Covid-19 pandemic in the range of a mid- to large-size natural catastrophe in terms of potential insured property and casualty claims. According to Autonomous (2020), “[i]ndustry insured loss estimates have shifted from ‘moderate hurricane’ equivalent to a potential full re-run of peak catastrophe years of 2011 or 2017 (> $100bn). … [W]e set a range of $31 [to] 86bn (1.3–3.6% of global premiums) for Covid-19 losses …The nature of these losses is unprecedented from a global accumulation perspective and in the ‘hard to model’ bucket.” Willis Towers Watson (2020) put the moderate to severe claims estimates in a similar range at USD 32–80 bn for the US and UK markets. However, at this early date, the range of uncertainty is very large, spanned by optimistic scenarios (USD 11 bn, WTW 2020) to worst-case scenarios (USD 140 bn, WTW 2020). Looking at the industry scenario estimates from WTW (2020) in their Fig. 10, the moderate case claims of USD 38 bn are predominantly driven by business interruption (USD 18.5 bn or 49%, of which approximately 10% are due to event cancelation).

To put these losses into context, Swiss Re (2020) estimates that natural catastrophe insured losses from two of the worst years in the last decade totaled USD 144 bn in 2017, driven by hurricanes Harvey, Irma and Maria, and USD 139 bn in 2011, primarily from a combination of the earthquakes in Japan and New Zealand and Thai flooding. While unpleasant and unexpected, these expected claims lie within the industry’s capacity.

### Business interruption

Given its high potential impact, business interruption insurance deserves more discussion. Business interruption insurance generally provides coverage for financial losses sustained as a result of an interruption caused by property damage; non-property damage business interruption coverage or extensions are not as prominently purchased and would generally have specific sublimits if purchased.

Pandemics are generally thought to have been excluded under property business interruption insurance or extensions as there is no direct property trigger. In coming up with their initial estimates, Autonomous (2020) states that, “[t]heoretically, the industry’s … economic losses should be low given the fact that, for the most part, they did not underwrite ‘pandemic’ disruption. However, the degree to which coverage may have been unwittingly provided is going to be severely tested.” The effectiveness of business interruption policy wordings in limiting losses is being challenged (WTW 2020), in particular interpretations of any limitations to physical damage trigger(s), explicit pandemic exclusions, and coverage for named perils only.

The potential impact of challenges to policy wordings could be very large and seems to have been underestimated by the insurance industry. See for instance Ralph (2020), GDV (2020a): While it was reported that add-ons to standard business interruption policies specifically covering infectious diseases were rare, a debate emerged around the question of whether certain types of non-property damage business interruption insurance, in particular policies covering restaurants or hotels, provided coverage in the case of a general lock-down.

Policy design varies across these coverages, so that general statements are not possible. But there are examples that indicate a significant need for improvement of wording. In Germany, for example, a common version of non-property business interruption coverage refers to a list of infectious diseases in the German Infection Protection Act (“Infektionsschutzgesetz,” IfSG). While the insurance industry argues that the Covid-19 losses are only covered if the current version of that list has explicitly become a part of the contract (see GDV 2020a), the argument can also be made that such losses are automatically included as these contracts refer to a dynamic list (see, e.g., Burghardt 2020). As the coronavirus was only added to the list at the end of January 2020, the courts’ positions in this legal controversy might have significant impact on the amount of losses ultimately paid as a consequence of the Covid-19 lock-down in Germany.

Some also argue that these coverages were meant for situations where an individual restaurant has to close because an employee has contracted an infectious disease and that a lock-down as a general prevention or containment measure would not be subject of such coverage (see for instance Ralph 2020, or GDV 2020a). Policies may have been written with only the individual scenario in mind, but apparently contract wording in many cases leaves room for legal interpretation. Clearly, for future coverage, policy wording must be improved in this area.

One can only speculate about why the accumulation risk associated with a pandemic in non-property damage business interruption and related products seems to have been underestimated. In addition to some of the behavioral biases mentioned earlier, another potential reason could be a “silo mentality” in underwriting, which would be consistent with the perception that pandemic risk as a source of accumulation risk was an issue in life, but not so much in property and casualty insurance.

### Other areas of “surprise”: public perception and regulatory restrictions

With the benefit of limited hindsight, two other areas may be characterized as “surprises” relative to the typical pre-Covid-19 scenario analysis. The first has to do with the public’s perception of the insurance industry. Normally, the perception of the insurance industry tends to improve following a natural catastrophe with large insured losses. However, the perception of the industry post-Covid-19 is likely to suffer for two reasons: first, some politicians may resort to socially charged language to justify the attempt to force retroactive coverage for business interruption claims, even in the face of clear contractual exclusions. Second, the uncertainty surrounding the interpretation of some, less clear exclusions will likely leave a trail of litigation as well as (politically charged?) conduct-related cases as the policies may not have performed neither as customers nor insurers expected.

Also, national and international regulators have been far quicker to impose capital management restrictions on, for example, bank and insurer dividends and share buybacks.^15^ In part this preemptive action was to ensure financial system stability but also in part to achieve a “socially acceptable risk sharing” of the pandemic’s impact. According to Svoronos and Vrbaski (2020), these “restrictions may contribute *to a more socially acceptable sharing of the overall costs of the pandemic”* (emphasis added). These restrictions were not limited to banks. Jones (2020) in Reuters reports that EIOPA, the European insurance supervisor, said that, “[i]nsurers and reinsurers in the European Union should temporarily suspend dividends and share buybacks, and consider postponing bonuses as well to ensure continuity in services during the coronavirus pandemic.”

## Lessons learned

### Future pandemic risk coverage

Based on the experience from Covid-19, there seems to be broad consensus in the insurance industry that the consequences of a pandemic cannot be covered in their entirety by the private insurance market (see, e.g., GDV 2020b). This has raised the issue of introducing public private partnerships that can provide protection against the financial impact of future pandemics. For instance, two programs that have been suggested in the US, are the *Business Continuity Protection Program* (BCPP), and a *Pandemic Risk Insurance Act* (PRIA). The latter proposal is based on the *Terrorism Risk Insurance Act* (TRIA) and would have the insurance companies take the first loss, with a federal backstop covering losses exceeding a threshold (see, e.g., Sclafane 2020). While this might be the right risk sharing mechanism for terrorism risk, its application to pandemic risk can cause severe incentive distortions and thus external moral hazard when the government has an interest in extending containment measures, as long as the resulting costs are covered by the insurers.

The BCPP proposal, which was developed jointly by the National Association of Mutual Insurance Companies (NAMIC), the American Property Casualty Insurance Association (APCIA) and Independent Insurance Agents & Brokers of America, Inc. (Big “I"), avoids this problem. Under this program, similar to the *National Flood Insurance Program* (NFIP), the insurance industry would not assume the risk but help administering protection on behalf of the government, which would provide the actual support through the *Federal Emergency Management Agency* (FEMA). According to the proposal, businesses could choose their desired level of protection for three months’ relief for up to 80% of payroll (excluding highly compensated employees), employee benefits and operating expenses. Rates would be calculated as a percentage of the payroll and expenses (see NAMIC 2020). Payments under the BCPP would be automatically paid in case of a federally declared public health emergency. The unambiguous trigger combined with the predefined amounts guarantees fast payment without any claims adjustment needed. This choice of trigger excludes the potential of (internal) moral hazard, but also leaves the insured with some basis risk resulting from the possibility that containment measures are only ordered on a local level.

As another example, the German Insurance Association’s proposal (GDV 2020b) for a pandemic support system highlights the industry’s expertise and the advantages of using existing customer relationships for a fast and efficient support payment process in future pandemics. Arguing that the financial consequences of a pandemic cannot be insured in the private sector, the GDV (2020b) green paper also emphasizes the importance of strong government involvement in financing a support system. The paper distinguishes between two models, one with flat rate levies and flat benefits (model A), and another one that would be more risk-based (model B). Acknowledging the potential adverse selection problem associated with a system that is not risk-oriented, it is argued that model A would have to be mandatory. As much as this argument is straightforward, this is an interesting proposal in so far as the German insurance industry in a similar discussion about whether flood insurance should be made mandatory has strongly argued against a compulsory system (see Schwarze and Wagner 2007).

Generally, as long as a support system is not designed to be mandatory, take-up can be a key question. Considering the generous government support that was provided during the Covid-19 crisis, charity hazard, or the “Samaritan’s dilemma,” might become an issue: If businesses anticipate significant ad hoc help from the government, this can crowd out voluntary protection programs (Raschky et al. 2013). The above-mentioned uncertainty about coverage under current policies in the Covid-19 crisis might add to a reluctance in the demand for pandemic insurance. Any protection program for future pandemic losses will have to be highly transparent with unambiguous policy triggers and should be communicated actively to the potential insured.

Regardless of the actual vehicle that is used to provide relief for business interruption losses or whether the government has a role in a protection program or not, pandemic catastrophe (cat) bonds may be used to support financing such relief. For instance, the German Insurance Association’s proposal for a pandemic support system (GDV 2020b) suggests to involve the capital markets via such bonds.

Cat bonds can help improving the efficiency of risk financing (see, e.g., Doherty and Richter 2002; Nell and Richter 2004). Among the reasons often cited are full collateralization and the potential of a fast claims process. *Pandemic* cat bonds are not a new concept. During the Covid-19 crisis, the World Bank’s pandemic cat bonds were triggered, providing funds to *International Development Association* (IDA) countries through the World Bank’s *Pandemic Emergency Financing Facility* (PEF) in order to help these countries in their response to the pandemic (Evans 2020).^16^ However, these bonds were heavily criticized for the complexity of the trigger mechanism and the resulting delay in payments (Baker 2020; Hartwig et al. 2020; Strohecker 2020). Reacting to this criticism, the World Bank which had initiated the PEF specifically to set up a way of getting funds to poor countries quickly, has now decided not to continue the program with a second iteration that had been expected for 2020 (Hodgson, 2020).

While the complexity issues associated with the World Bank’s pandemic cat bonds may have to do with the specific focus of the PEF, simple and transparent triggers will generally be necessary to ensure acceptance of such instruments. In order to limit incentive distortions, parametric triggers will be helpful (Doherty and Richter 2002; MacMinn and Richter 2018). As we have seen, the question of an adequate definition of triggers has been a problem for many insurance products as well. If, however, this problem can be solved in a pandemic support system for non-property damage business interruption losses as discussed above, there is no reason why it would not be possible to use these or similar triggers in designing a cat bond that helps financing this support.

A standard reason for the use of cat bonds is that they are particularly attractive from an investor’s perspective, assuming the securitized catastrophe risk has low correlation with market risk (see Litzenberger et al. 1996). This argument is of limited validity for extreme catastrophes (Gürtler et al. 2016) and may thus be questionable for pandemic cat bonds (note, however, that during the Covid-19 crisis, stock markets rebounded relatively fast). Still, such a bond, in particular a cat bond issued by the government (sovereign cat bond) could be an efficient risk allocation tool for highly correlated losses: It provides a means to atomize the risk and ex ante organize the distribution of losses across a large number of individuals. This, though, is only possible if the cat bond is available not just for institutional investors, but also in particular for individual investors. In order to make this possible, it would be advisable to limit an individual’s loss, for instance to the amount of the coupon. In addition, these investments could be tax-advantaged in order to set further incentives.

### Insurer crisis risk management

Crisis scenarios offer an opportunity for risk managers to reflect upon and adapt their ERM framework and for policy-makers and regulators to update their “response playbook” in light of the unique “lessons learned” from the crisis.

In terms of risk management, the lessons learned from the Covid-19 crisis for insurance risk managers include, first, that as a long-term investor, insurers should build resilience upfront in the form of sufficient capital and liquidity resources combined with limiting risk accumulations to be consistent with those resources. This is because executing contingency plans during a crisis often comes at a high cost and typically cannot fully recover to pre-crisis conditions. Second, scenario analysis is important for both setting and assessing risk appetite in a dynamically changing environment. Nonetheless, scenario analysis can be improved by using reverse stress tests to challenge correlation assumptions. For example, most maximum probable loss scenarios for entertainment/event cancelation assumed diversification, leading to a few large losses. In contrast, answering the question, “What conditions would lead to a full limit loss on substantially all of the portfolio?” would be an illuminating exercise in light of the Covid-19 crisis. Furthermore, scenario analysis can be improved by testing exposure to contract wording challenges and further considering how public perception may change when wordings are challenged.

With regard to the insurability of pandemic risk, the industry has learned that business interruption is not an insurable risk if it is caused by containment activities to manage a global pandemic. In this regard, insurers need to improve policy wordings, focusing on increased harmonization, transparency, and enforceability. In addition, they need to re-underwrite renewals for the book immediately to reflect the new wordings and carefully consider whether these lessons are also valid by analogy for other lines such as cyber insurance which may be affected by large, systemic accumulations.

## Footnotes

1 For a brief discussion of building insurer resilience to financial crises see, for instance, Berliet (2009); for more general discussions on company resilience towards natural disaster risk see, e.g., Berke and Campanella (2006), or Fuchs (2009), and for the context of cyber risk Hofmann et al. (2018).

2 The term *external moral hazard* refers to insurance-induced changes in behavior of third parties which affect the size of insurance payments (see Nell 1993; Nell et al. 2009).

3 In our discussion of insurability, we loosely follow a set of criteria that goes back to Walter Karten (see, for instance, Karten 1972, 1997 or Karten et al. 2018): We look for potential insurability problems resulting from correlation and/or the size of a loss, information asymmetries (in particular moral hazard), insufficient knowledge about the loss distribution and difficulties in precisely defining the insured risk.

4 See Hartwig et al. (2020) for a detailed discussion about the effect of the correlation of pandemic losses on the amount of capital that is needed to credibly insure the risk.

5 https://bizfluent.com/info-8327367-risk-mitigation-vs-contingency-planning.html and https://simplicable.com/new/risk-management-vs-contingency-planning (accessed 3 August 2020).

6 There is some evidence that, while a more secure firm may have to hold a higher quantum of capital, the cost of that capital decreases because it is in fact more secure. Evidence for the cost of capital for financial services firms negatively correlated with the firm’s rating can be found in Wilson (2015).

7 A logical response to uncertainty surrounding the event space and/or probability distribution is to invest in more information and to learn from past experiences. Doherty et al. (2015) conclude that brokers can help ex post in distinguishing between ex ante unknown risks that are insurable and should be insured retroactively and into the future and those that are not.

8 As an interesting aside, based on similar scenario results Allianz decided a decade ago against issuing a pandemic linked security. This decision was taken based on the hypothesis that similar expenditures on out of the money equity put options would be just as effective during a pandemic scenario but also respond favorably to more mundane and frequent financial market crisis.

9 Allianz did an ad hoc corona virus scenario in mid-January 2020, calibrated between the SARS and 1918 Spanish influenza historical scenarios. Paraphrased, the conclusions were that the overall impact of a worst-case scenario was deemed material and driven by financial market developments but the outcome was within risk appetite defined by other comparable financial market scenarios. The combined impact from LH/PC insurance claims was estimated to be materially less than the losses from financial markets with the greatest impact in PC anticipated from business continuity.

10 Chang et al. (2004) comment in the context of Taiwan: “At the peak of the SARS epidemic, significant reductions in ambulatory care (23.9%), inpatient care (35.2%), and dental care (16.7%) were observed.” Note that a lower uptake of non-essential medical procedures may cause higher essential procedures in the future to the extent that the non-essential procedures were preventative. In any case, it is likely that the backlog of non-essential procedures would merely be delayed rather than eliminated.

11 Enz (2000) puts forth a model and empirical evidence to support an income or wealth effect related to country GDP for insurance penetration rates, with lower income countries having lower penetration rates.

12 While this is not documented for the 1918 Spanish influenza pandemic, more recent research points in this direction, with Jaffe et al. (2020), “[o]ur findings thus imply that high-income countries are better placed to face this pandemic, probably due to a higher availability of running water and soap, hospital beds, qualified medical personnel and/or technical equipment. In contrast, low-income countries seem more vulnerable to COVID-19 since they are likely to experience higher mortality rates than countries with better and more accessible services.”

13 See the Solvency II Directive (EU Directive 2009/138/EC), Articles 87–89 and 100–108: Solvency II own funds consist of basic own funds and ancillary own funds. Basic own funds are calculated as the excess of liabilities over assets, plus subordinated liabilities. Ancillary own funds are “items other than basic own funds which can be called up to absorb losses” (Article 89 of the EU Directive 2009/138/EC). The SCR under Solvency II is the amount of basic own funds an insurer needs to hold in order for its survival probability to be at least 99.5%. In other words, the SCR represents the 99.5% Value at Risk of the basic own funds. Insurers can either use a standard formula or an internal model to calculate the SCR. Regarding the specific topic of this paper, it can be noted that the standard formula for life insurers includes a catastrophic shock scenario that was developed with a focus on pandemic risk (see Kraut and Richter 2014).

14 In the case of Allianz’s Q1:2020 published financial results, there was a decrease in Solvency II own funds of EUR −7.9 bn as asset values fell and liability values increased, compared to a capital increase of EUR + 1.8 bn reflecting the more uncertain credit environment and being closer to the guarantees.

15 Svoronos and Vrbaski (2020) present an overview of supervisory initiatives by jurisdiction in their Table 2.

16 Securities designed as instruments to hedge extreme mortality changes for life insurance books of business have been issued for a while, starting with Swiss Re’s Vita Capital transaction in 2003 (see, e.g., MacMinn and Richter 2018).
